# A Randomized Controlled Trial to Assess the Use of a Virtual Decisional Aid to Improve Knowledge and Patient Satisfaction in Women Considering Breast Reconstruction Following Mastectomy

**DOI:** 10.7759/cureus.12018

**Published:** 2020-12-10

**Authors:** Lee Varelas, Francesco M Egro, Nicole Evankovich, Vu Nguyen

**Affiliations:** 1 Plastic Surgery, University of Pittsburgh, Pittsburgh, USA

**Keywords:** breast cancer, breast reconstruction, decisional aid, mastectomy, virtual decisional aid, patient knowledge, patient satisfaction, surgeon satisfaction

## Abstract

Background

The decisional process of navigating breast reconstruction surgery is very challenging for patients with a breast cancer diagnosis. This study aims to assess the impact of a virtual breast reconstruction decision aid program on the decision-making process of breast cancer patients considering breast reconstruction.

Methods

A two-arm, randomized, controlled trial was conducted at the University of Pittsburgh. Patients were blindly assigned to one of two arms: Emmi Decide (Emmi Solutions LLC, Chicago, IL) program prior to traditional consultation (intervention) and traditional consultation alone (control). All patients completed a baseline pre- and post-intervention questionnaire to evaluate knowledge, patient satisfaction, and psychological status. Surgeons' satisfaction and consultation time were also recorded.

Results

A total of 26 patients participated in the study (n=13 in each arm). Patients in the intervention group reported a greater BREAST-Q reconstruction module score (control=47.9±8.2, intervention=56.8±4.2, *p*=0.0017), lower decisional conflict scale score (control=30.2±11.8, intervention=14.5±8.8, *p*=0.017), and improved patient knowledge (control=70.8±15.5%, intervention=83.1±13.8%, *p*=0.018). No difference was noted in consultation time (control=51.0±7.8 min, intervention=47.8±13.7 min, *p*=0.46) and psychological testing (control=49.7±16.0, intervention=44.6±15.2, *p*=0.26). However, surgeons reported greater satisfaction with their consultations with interventional group participants (control=3.4±0.7, intervention=4.8±0.4, *p*=0.000056).

Conclusions

The use of a virtual decisional aid program to assist the decision-making of breast reconstruction patients was shown to significantly benefit both patients and surgeons, by improving patient knowledge and satisfaction without placing an additional psychological burden on them. This supports the notion that this resource is a promising tool that can improve the difficult process of breast reconstruction in the vulnerable population of breast cancer patients.

## Introduction

The decisional process of navigating breast reconstruction surgery is difficult for most patients but is especially challenging for patients with an active breast cancer diagnosis as detailed by existing studies that demonstrate the high prevalence of anxiety and stress in cancer patients considering breast reconstruction [1]. Studies have also shown that breast reconstruction can alleviate some of the psychological stresses associated with the process [2]. However, given the vulnerable psychological state of this particular patient population, shared decision making is crucial in order to allow patients to make informed decisions that align with their personal values and goals. Moreover, shared decision making has been determined to be viewed favorably by both patients and surgeons, often correlating with improved surgical outcomes [3].

Decisional aids are educational tools that are becoming increasingly popular in order to reduce this difficulty in patients. Several studies have shown their value in aesthetic breast reconstruction populations [4-6]. However, only few randomized studies exist in breast cancer patient populations [7,8].

The Emmi Decide (Emmi Solutions LLC, Chicago, IL) series of programs are new decisional aids that aim to educate viewers about condition-specific treatment options, to explain the corresponding risks and benefits of each treatment option, and to encourage patients to better understand their goals and preferences throughout the treatment process. The Breast Reconstruction program was designed specifically to educate women who will have or have had a mastectomy to treat breast cancer. It does so by providing information regarding the basic anatomy of the breast, available reconstructive procedures, and other considerations specific to breast reconstruction.

Quality initiatives that improve the patient experience are becoming increasingly sought after [9]. While there have been studies investigating the use of decisional aids in aesthetic plastic surgery populations, there are very few available that specifically target breast cancer reconstruction patients. Furthermore, the Emmi Decide program has yet to be quantitatively assessed and we believe this study will show the value of Emmi Decide in improving the patient experience. Additionally, evidence showing that the use of decisional aids reduces the duration time of pre-surgical consultations will help recruit other surgeons to participate in the program, further bolstering patient experience improvement.

With this in mind, this study aims to assess the impact of a new virtual breast reconstruction decisional aid program on the decision-making process of breast cancer patients considering breast reconstruction by evaluating patient knowledge, patient satisfaction, and consultation time. The hypothesis is that a decisional aid program will help patients' knowledge and satisfaction.

## Materials and methods

Participants

Eligible patients included English-speaking adult women >18 years that had been diagnosed with breast cancer (stage I or II only) that had been advised to undergo or had already undergone a mastectomy. The exclusion criteria were patients with metastatic disease, cognitive impairment, or uncontrolled psychiatric diagnosis, who had previously undergone breast reconstructive surgery or had previously consulted with another plastic surgeon, and a history of radiation therapy.

Setting and study

**Figure 1:**
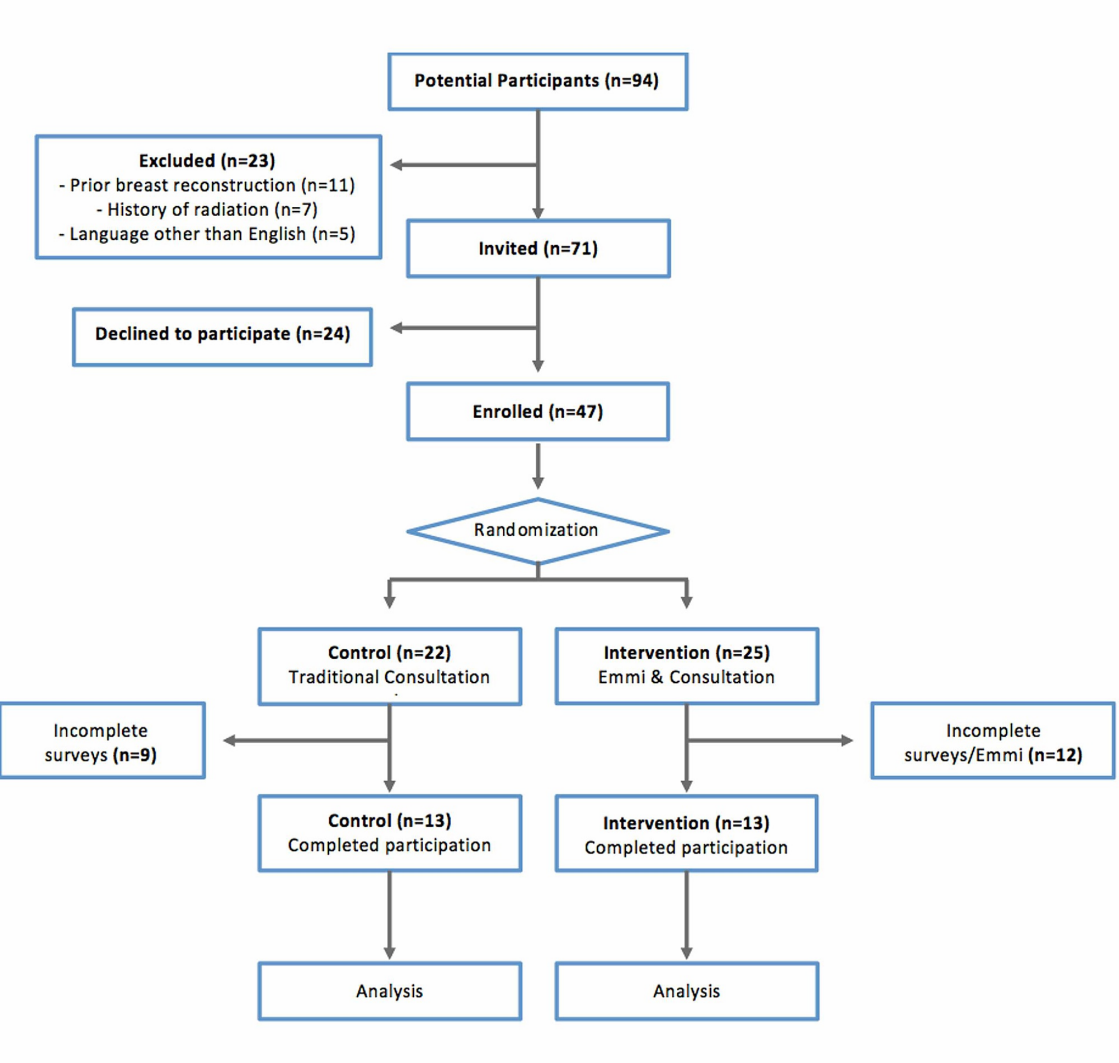
Flowchart outlining a summary of the study’s methodology including the number of patients that were enrolled, randomized, and ultimately included in the study.

The study was approved by the University of Pittsburgh Institutional Review Board (PRO 17110382). A two-arm, parallel, randomized, controlled, single-surgeon trial was conducted at the University of Pittsburgh Medical Center. Patients were recruited from a single outpatient plastic surgery clinic from January 2019 until December 2019, when the University of Pittsburgh Medical Center stopped utilizing the virtual decisional aid being investigated. Each patient’s participation, including recruitment, study completion, and follow-up, occurred within a two-week period. Patients were blindly assigned to one of the two arms (Figure 1): Emmi Decide program prior to traditional consultation (intervention) and traditional consultation alone (control). A single study coordinator enrolled participants, randomized participants using a random number generator with a 1:1 ratio allocation, and provided each arm’s participants the relevant materials for their participation. The study’s surgical team were blinded to patient allocation. All patients completed a baseline questionnaire prior to randomization, as well as a follow-up survey immediately following the intervention and/or consultation. A single senior surgeon completed a surgery satisfaction survey for each patient, also documenting the duration of the consultation. 

Research groups

Intervention Group

Patients allocated to the intervention group were provided the Emmi Decide program to view during the week prior to their scheduled traditional consultation. This resource took approximately 20 minutes to complete. Once they completed the Emmi Decide program they attended a traditional consultation with their plastic surgeon, before completing the follow-up survey.

Control Group

Patients allocated to the control group attended a traditional consultation before completing a follow-up survey.

Patient survey

Each patient completed two identical surveys. The baseline survey was completed prior to their consultation and a follow-up survey was completed following their consultation. Each survey consisted of the following four sections:

1. Knowledge assessment test: This structured knowledge assessment test consisted of five items and addressed general breast cancer information, surgical and reconstructive knowledge pertinent to available breast reconstruction procedures, and consequent treatment benefits and complications.

2. State-Trait Anxiety Inventory (STAI) scale: This scale consisted of six items and has been validated to assess for psychological status. This scale is scored between 20 and 80, with a larger value correlating to higher anxiety levels.

3. Decisional conflict scale: This scale consisted of 16 items and has been validated to assess the extent to which an individual experienced conflict regarding their surgical decision [10]. When considering the decisional conflict scale, scores are tallied from 0 to 100, with a larger value correlating to higher decisional conflict [11]. This scale was used to quantify patient satisfaction, as the lower conflict in one’s decision is considered indicative of greater satisfaction in the patient’s breast reconstruction choice.

4. BREAST-Q reconstruction module: This scale consisted of 15 items that were taken from the BREAST-Q reconstruction post-operative module, which is used to assess satisfaction with information provided by a patient by their surgeon. This module was also used to quantify patient satisfaction and is scored between 15 and 60, with a higher value correlating to better patient satisfaction.

Surgeon survey

A survey was completed by the senior surgeon of the surgery team that conducted the consultation with enrolled patients, immediately following their scheduled consultation. The survey examined the time of consultation and surgeon satisfaction by mean of a five-item survey, scored using a five-point Likert scale. This survey addressed surgeon satisfaction with patient confidence, patient knowledge, and preparedness for the consultation, as well as their satisfaction with the duration of the consultation.

Outcomes

Primary Outcomes

Two primary outcomes were selected: (1) patient satisfaction, by means of the decisional conflict scale and the BREAST-Q reconstruction module; and (2) patient knowledge, by means of a short knowledge assessment test.

Secondary Outcomes

Three secondary outcomes were selected: (1) psychological status, by means of the STAI scale; (2) surgeon satisfaction, by means of a surgeon satisfaction survey; and (3) time of consultation, by means of a surgeon satisfaction survey.

Statistical analysis

All data were analyzed using Excel (Microsoft, Redmond, WA). Descriptive statistics were used to compare baseline characteristics, knowledge questionnaires, and satisfaction scores among groups. Student paired t-test was used to compare the means of the two groups. Statistical significance was considered to be a value of *p*<.05; p-values are mentioned or described only if results are statistically significant.

## Results

A total of 26 female participants diagnosed with stage I or II breast cancer were randomly allocated to either the intervention (n=13) or the control group (n=13). A summary of the results can be seen in Table 1.

**Table 1:** Comparison of control and intervention groups results. *Statistically significant results noted with an asterisk. SD, standard deviation; %, percentage; n, number; STAI: State-Trait Anxiety Inventory

	Control (n=13)	Intervention (n=13)	p-Value
Age, mean ± SD	52.1 ± 13.0	47.1 ± 9.0	0.13
Cancer stage, n (%)			
I	7 (53.8)	7 (53.8)	
II	6 (46.2)	6 (46.2)	
STAI scale, mean ± SD	49.7 ± 16.0	44.6 ± 15.2	0.26
Decisional conflict scale, mean ± SD	30.2 ± 11.8	14.5 ± 8.8	0.017*
BREAST-Q reconstruction module, mean ± SD	47.9 ± 8.2	56.8 ± 4.2	0.0017*
Pre-consultation knowledge test (%), mean ± SD	61.5 ± 19.1	67.7 ± 19.2	0.20
Post-consultation knowledge test (%), mean ± SD	70.8 ± 15.5	83.1 ± 13.8	0.018*
Surgeon satisfaction scale, mean ± SD	3.4 ± 0.7	4.8 ± 0.4	0.000056*
Consultation time (min), mean ± SD	51.0 ± 7.8	47.8 ± 13.7	0.46

The mean age was 47 years for the interventional group (range from 32 to 62) and 52 for the control group (range from 32 to 75). Both control and intervention groups were identical when considering cancer staging, with 53.8% of patients diagnosed with stage I breast cancer and 46.2% diagnosed with stage II breast cancer in each arm.

Pre-consultation knowledge assessment scores were comparable between the two groups (control=61.5±19.1%, intervention=67.7±19.2%, *p*=0.20). Following consultation, knowledge assessment scores improved in both groups but were significantly improved in the intervention group (control=70.8±15.5%, intervention=83.1±13.8%, *p*=0.018).

The post-consultation STAI scale score was higher in the control group but did not reach statistical significance (control=49.7±16.0, Intervention=44.6±15.2, *p*=0.26), implying that the additional decisional aid tool had no effect on the psychological status of patients.

Two surveys, a decisional conflict scale and a BREAST-Q reconstruction module were utilized to evaluate patient satisfaction. When considering the decisional conflict scale, post-consultation scores were significantly lower in the intervention group than in the control group (control=30.2±11.8, intervention=14.5±8.8, *p*=0.017). When comparing post-consultation scores for the BREAST-Q reconstruction module, the intervention group scored significantly greater than the control group (control=47.9±8.19, intervention=56.8±4.19, *p*=0.0017).

Surgeon satisfaction was also significantly higher in the intervention group when compared to the control group (control=3.4±0.7, intervention=4.8±0.4, *p*=0.000056). Meanwhile, consultation time was lower in the intervention group but did not reach statistical significance (control=51.0±7.8 min, intervention=47.8±13.7 min, *p*=0.46). No participants were exposed to harm and there were no unintended effects from involvement in the study.

## Discussion

Decisional aids have been found to be valuable tools that help improve the patient experience undergoing breast reconstruction [7,8]. Sherman et al. investigated the effect of one such decisional aid, BRECONDA, on breast patients eligible for reconstruction following mastectomy, and found that one-month decisional conflict was significantly lower in the intervention group compared to the control group [8]. However, the Emmi Solutions series of decisional aids has not been investigated in the breast cancer population. This study intended to investigate the effect of a virtual decisional aid (Emmi Decide breast reconstruction series), on patient satisfaction and knowledge acquisition in breast cancer patients. Secondary outcomes that were also considered included the effect of the decisional aid on patient psychological status, consultation duration and surgeon satisfaction.

Participants were initially randomly allocated to the two arms of the study, but all patients took identical baseline surveys. The purpose of this baseline survey was to ensure there was no baseline difference between the two study arms in any of the outcomes to be investigated. Both arms scored similarly on the baseline survey, and no difference was noted in any area of the survey. This provided confidence that any difference in follow-up survey scores found between the two study arms, would primarily be due to the intervention and not any pre-existing variables.

The mean age of all patients was 50 years, however, the intervention group was younger and had a narrower range (mean age=47, range from 32 to 62) compared to the control group (mean age=52, range from 32 to 75). Older patients were less likely to elect to participate and were also more likely to leave components of the study incomplete. We believe this observed difference in age between arms is likely due to the role technology plays in the study’s methods. Younger patients likely felt more comfortable with the online platform of the Emmi Decide decisional aid and thus were more likely to engage with the study and complete all components. Other studies have shown that age does play a role in a patient’s likelihood to receive information on breast reconstruction. For instance, Frisell et al. demonstrated that older women were significantly less likely to receive information regarding immediate breast reconstruction and that age was an independent predictor of not receiving relevant information [12]. Accordingly, these differences in age between groups should be noted when considering the generalization of the results to the wider breast cancer population.

To evaluate patient satisfaction, two surveys were used. The decisional conflict scale has been validated in prior studies to quantify patient satisfaction, as greater confidence in one’s decision is considered indicative of greater patient satisfaction [13]. The results of this study showed that patients that utilized the Emmi Decide decisional aid prior to their traditional consultation scored better on this scale than the control group (control=30.2±11.8, intervention=14.5±8.8, *p*=0.017). This supports the hypothesis that the use of this particular decisional aid would improve patient confidence in challenging breast reconstruction decisions. Similarly, the intervention group scored significantly better on the BREAST-Q reconstruction module (control=47.9±8.2, intervention=56.8±4.2, *p*=0.0017), demonstrating a higher level of satisfaction with medical information provided to them. Combined, these results overwhelmingly demonstrate that the use of a decisional aid to complement traditional consultation in breast cancer populations can improve patient satisfaction compared to traditional consultation alone.

Patients in the intervention group also exhibited greater comprehension of general breast cancer information and reconstruction benefits and complications, scoring significantly better on the five-item, post-consultation knowledge test when compared to the control group (control=70.8±15.5, intervention=83.1±13.8, *p*=0.018). While this portion of the survey was self-written by the study team and is not yet validated by outside sources, it still demonstrates that the use of a decisional aid can improve a patient’s understanding of fundamental information pertinent to their care.

Surgeon satisfaction was overwhelmingly higher in the intervention group (control=3.4±0.7, intervention=4.8±0.4, *p*=0.000056). This is critical as the successful widespread use of a decisional aid in this population will only be possible if supported by the surgeons that would be needed to implement it within their practices. While evidence that the decisional aid significantly improved patient satisfaction and knowledge should satisfy most surgeons, implementation would undoubtedly be bolstered by the added benefit of satisfaction on the clinician’s end as well.

Another incentive for surgeons to utilize this resource would be if the decisional aid helped to improve efficiency in the clinic. We hypothesized that the use of the decisional aid would also decrease consultation time, reasoning that patients that were more prepared and knowledgeable would need less clarification in person by their surgeons. The results did find that that consultation time trended lower in the intervention group compared to the control group (control=51.0±7.8 min, intervention=47.8±13.7 min, *p*=0.46), but this difference was not found to be statistically significant. Anecdotally, the clinicians involved in the study noted an increased ability to discuss a broader range of reconstructive options and/or to delve deeper into these available options, while at the same time not increasing the overall length of the consultation.

Finally, psychological status was also assessed by use of the STAI scale. While the intervention group did have a lower mean score than the control group (control=49.7±16.0, intervention=44.6±15.2, *p*=0.26), no statistically significant difference was observed between the two groups. This implies that the use of the decisional aid did not meaningfully impact a patient’s psychological status regarding the process. Given the gravity of a breast cancer diagnosis, it is very reassuring that the virtual decisional aid did not worsen anxiety while providing benefit in the areas of patient satisfaction and knowledge, as well as surgeon satisfaction.

This study faced a number of limitations. Most patients who were asked to partake in the study (n=71) acknowledged the importance of the venture, however, 24 patients elected not to participate citing that they were overwhelmed with their cancer prognosis and did not wish to partake in any intervention that was not essential to their care. Additionally, we were also forced to remove 21 patients from the study due to incomplete participation and when we considered the typical volume of breast cancer patients, we did not take into account the number of patients that would be excluded from the study (n=23). Finally, during the study period, the University of Pittsburgh Medical Center transitioned educational resources away from Emmi Decide, which resulted in the termination of further recruitment of patients. However, while sample size may have been a limiting factor, the study still produced statistically significant results in both primary outcomes and one secondary outcome. Age likely plays a role in whether these results can be generalized to the greater breast cancer population given the lower participation among the older population. While the Emmi Decide decisional aid has been designed to be easy to use for participants of all computer skill levels, it does rely on a basic understanding of technology as well as the ability for patients to access the platform, which in certain patients was an issue. Another limitation of the study is that the population being investigated is a particularly vulnerable group of patients with a high level of stress and anxiety, which might have influenced the satisfaction and psychological scales. Future research should focus on evaluating other decisional aid programs as well as understanding long term satisfaction outcomes of patients.

## Conclusions

In conclusion, a virtual decisional aid utilized prior to breast reconstruction consultations demonstrated a benefit to both patients and surgeons in a prospective, randomized model. Its use significantly improves patient knowledge, decisional confidence, and satisfaction. In addition, surgeon satisfaction was also significantly improved. These measures were demonstrated without compromising the patient’s psychological status or consultation time. Given how vulnerable this particular patient population can be, the ease and cost of implementing this tool is a promising way to build patient confidence, advance patient understanding and alleviate some of the stress involved with the difficult process of breast reconstruction.
